# An analysis of the association between breast density and body mass index with breast cancer molecular subtypes in early breast cancer: data from a Spanish population

**DOI:** 10.1007/s12094-024-03469-6

**Published:** 2024-05-11

**Authors:** Isabel Calvo, Marta González-Rodríguez, Fernando Neria, Isabel Gallegos, Lourdes García-Sánchez, Rosa Sánchez-Gómez, Silvia Pérez, María Fernanda Arenas, Laura G. Estévez

**Affiliations:** 1 Breast Cancer Unit- Oncology, MD Anderson Cancer Center, Madrid, Spain; 2 Fundación MD Anderson Internacional España, Madrid, Spain; 3 Facultad de Medicina, Universidad Francisco de Vitoria, Madrid, Spain; 4 Breast Cancer Unit- Oncology, Hospital de Segovia, Segovia, Spain; 5 Breast Cancer Unit- Oncology, Hospital de San Pedro, Logroño, Spain; 6 Breast Cancer Unit- Radiology, MD Anderson Cancer Center, Madrid, Spain

**Keywords:** Body mass index, Obesity, Mammographic breast density, Early breast cancer

## Abstract

**Purpose:**

Breast cancer is an important health problem, like obesity and dyslipidemia, with a strong association between body mass index (BMI) and breast cancer incidence and mortality. The risk of breast cancer is also high in women with high mammographic breast density (MBD). The purpose of this study was to analyze the association between BMI and MBD according to breast cancer molecular subtypes.

**Methods:**

This transversal, descriptive, multicenter study was conducted at three Spanish breast cancer units from November 2019 to October 2020 in women with a recent diagnosis of early breast cancer. Data were collected at the time of diagnosis.

**Results:**

The study included 162 women with a recent diagnosis of early breast cancer. The median age was 52 years and 49.1% were postmenopausal; 52% had normal weight, 32% overweight, and 16% obesity. There was no association between BMI and molecular subtype but, according to menopausal status, BMI was significantly higher in postmenopausal patients with luminal A (p = 0.011) and HER2-positive (p = 0.027) subtypes. There was no association between MBD and molecular subtype, but there were significant differences between BMI and MBD (p < 0.001), with lower BMI in patients with higher MBD. Patients with higher BMI had lower HDL-cholesterol (p < 0.001) and higher insulin (p < 0.001) levels, but there were no significant differences in total cholesterol or vitamin D.

**Conclusions:**

This study showed higher BMI in luminal A and HER2-positive postmenopausal patients, and higher BMI in patients with low MBD regardless of menopausal status.

## Introduction

Breast cancer continues to be an important health problem and is one of the most common causes of cancer deaths in women worldwide, with an estimated 358,967 new cases and 90,665 breast cancer-related deaths in the European Union annually [1].

Although genetic profiling, age of menarche and menopause, parity, age of the first child, previous occurrence of cancer, and breast density are all well-known risk factors for breast cancer, lifestyle is considered an increasingly important, modifiable contributing factor to breast cancer etiology [2].

Obesity, defined as a body mass index (BMI) of ≥ 30 kg/m^2^, affects over 600 million adults worldwide, and the World Health Organization estimates that 40% of adult women are overweight (BMI of ≥ 25 kg/m^2^), with the prevalence tripling between 1975 and 2016 [3]. The prevalence of obesity varies widely by country, with low rates in countries such as Vietnam (2.1%) or Japan (4.4%) compared with 37.3% in the United States and the highest rates in Oceania (Nauru 61%, Cook Islands 55%) [1]. Except for some regions in sub-Saharan Africa and Asia, more people are now obese than underweight [1].

Several studies [4–6] have shown a significantly strong association between increased BMI and higher breast cancer incidence and specific mortality in postmenopausal women. However, in premenopausal women, high BMI is associated with a reduced risk of breast cancer [7].

The precise mechanisms whereby obesity plays a protective role against breast cancer in premenopausal women, but represents a risk factor after menopause, remain elusive [2].

Furthermore, two meta-analyses described that, in premenopausal women, obesity is associated with high-risk estrogen receptor (ER)-negative and triple-negative breast cancer but, in postmenopausal women, obesity seems to be a risk for hormone receptor-positive breast cancer [8, 9]. However, another meta-analysis that studied the association between obesity, hormone receptor, and menopausal status, reported an increased hazard ratio for overall survival in heavier versus lighter women independently of hormone receptor or menopausal status [10]. Consequently, more studies are currently needed to elucidate the role of obesity in different breast cancer subtypes.

Mammographic breast density (MBD) is based on the proportion of stromal, epithelial, and adipose tissue in the breast. MBD is also an independent risk factor for the development of breast cancer, with a higher risk in women with high density. A systematic review and meta-analysis of 42 studies found that the relative risk of incidental breast cancer is 2.92 for women with heterogeneously dense breasts (type C) and 4.64 for women with extremely dense breasts (type D), compared to women with almost entirely fatty breasts (types A and B) [11].

MBD is influenced by factors such as age and BMI (MBD decreases with increasing age and BMI), and increases with hormone replacement therapy [12] Therefore, there is a possible paradox in the relationship between breast cancer risk and fat tissue depending on its localization (high risk for body fatness but not for breast adipose tissue) [13].

Fat tissue has been described as a microenvironment promoting carcinogenesis through different mechanisms, in particular, chronic inflammation [14], but it also has a potentially protective role, especially as a source of vitamin D [13, 15]. Furthermore, recent studies have shown that increased levels of leptin and decreased adiponectin secretion are directly associated with breast cancer development [2].

Dyslipidemia is strongly associated with obesity and has been independently linked with breast cancer risk and survival [16], but data are conflicting. The ACALM study demonstrated that women aged above 40 years with high cholesterol were 45% less likely to develop breast cancer than women with normal cholesterol levels [17]. Moreover, some studies observed that low HDL-cholesterol was associated with higher estrogen levels and absolute mammographic density (both independent risk factors for breast cancer) [18]; and intratumor cholesteryl ester accumulation was associated with more aggressive tumors, including grade 3, HER2-positive, and triple-negative breast cancers [19]. However, based on the results of recent studies, 27-OH-cholesterol is potentially a better biomarker than total cholesterol [2].

Vitamin D is known for its anti-cancer properties, including induction of apoptosis and inhibition of angiogenesis and metastasis [20]. Low vitamin D levels were shown to be associated with increased overall and disease-specific breast cancer mortality [20, 21]. Furthermore, vitamin D deficiency increased the risk of recurrence of luminal breast cancer, but this relationship was not found in patients with HER2-positive or triple-negative cancer subtypes [22].

Hyperinsulinemia is an independent risk factor for poor breast cancer prognosis and is associated with low adiponectin levels and shorter breast cancer survival [23]. Moreover, elevated HOMA-IR scores and low adiponectin levels are both associated with obesity and increased breast cancer mortality. However, in premenopausal women, high circulating insulin levels may protect against breast cancer, the same as obesity [24].

The objectives of the current study were 1) to analyze the association between BMI and MBD with breast cancer molecular subtypes and 2) to study the possible differences between cholesterol, vitamin D, and insulin levels in recently diagnosed early breast cancer.

## Methods

The study included women with a recent diagnosis of early breast cancer during a 1-year period at three Spanish breast cancer units (MD Anderson Cancer Center Madrid, Segovia Hospital, and San Pedro Hospital of Logroño).

Oncologists at the breast cancer units completed a questionnaire at diagnosis of all included women about lifestyle (e.g., diet, exercise, smoking habit). Clinical characteristics (hypertension, diabetes, menopausal status, breast density, weight, height, and abdominal size) and tumor characteristics (TNM, estrogen receptors [ER], progesterone receptors [PR], Ki67, and HER2) were recorded. Blood tests for total cholesterol, LDL-cholesterol, HDL-cholesterol, triglycerides, insulin, and vitamin-D (25-OH vitamin D) were conducted at diagnosis.

Exercise was recorded as a ‘Yes/No’ response regarding whether the subject completed more than 150 min per week of moderate exercise (OMS recommendations). Diet was studied by collecting information about fruit and vegetable consumption, weekly alcohol consumption, olive oil used, and processed foods intake. Breast density information was obtained from the breast radiology report. Tumors were classified, according to the 13th St Gallen International Breast Cancer Panel, into luminal-A like (ER/PR positive, Ki-67 < 20%), luminal-B like (ER/PR positive, Ki-67 ≥ 20%), HER2-positive (ER and PR positive/negative*,* HER2-positive) and triple-negative (ER-, PR-, and HER2-negative).

The study received the approval of the hospital MD Anderson Cancer Center, all data of patients were coded and do not suppose any risk to the integrity of the patients.

All statistical analyses were performed with R software, version 4.1.1. Quantitative variables were described as median [IQR] and qualitative variables as absolute (n) and relative (%) frequencies. Chi-square or Fisher test were used to evaluate significant differences between qualitative data. A non-parametric Kruskal–Wallis rank sum test was used to evaluate differences in quantitative variables (total cholesterol, LDL-cholesterol, HDL-cholesterol, triglycerides, insulin, and vitamin-D) within MBD or molecular subtype subpopulations; postmenopausal and premenopausal differences in each MBD or molecular subtype subpopulation were evaluated by non-parametric Wilcoxon rank sum test. Correlations between BMI and blood test variables were determined by Spearman rank correlation coefficient (ρ). Differences with a p-value ≤ 0.05 were considered to be statistically significant.

## Results

### Patient characteristics

Overall, 162 women with a recent diagnosis of early breast cancer were included in the study at three Spanish breast cancer units from November 2019 to October 2020. The patient characteristics are shown in Table 1. The median [range] age was 52 [46–62] years and 49.1% were postmenopausal.

**Table 1:** Patients’ characteristics

	*N*	Overall, *N* = 162^a^	Normal BMI (18.5–24.9 kg/m^2^) *N* = 84 (52%)^a^	Overweight BMI (25–29.9 kg/m^2^) *N* = 52 (32%)^a^	Obese BMI (≥ 30 kg/m^2^) *N* = 25 (16%)^a^
**Age (years)**	159	52 [46–62]	50 [44–57]	54 [48–62]	60 [55–66]
**Race**	161				
Caucasian		157 (97.5%)	81 (96.4%)	51 (98.1%)	25 (100.0%)
Hispanic		3 (1.9%)	2 (2.4%)	1 (1.9%)	0 (0.0%)
Asian		1 (0.6%)	1 (1.2%)	0 (0.0%)	0 (0.0%)
**Hypertension**	161	31 (19.3%)	8 (9.5%)	11 (21.2%)	12 (48.0%)
**Menopause**	161				
Pre-menopause		82 (50.9%)	52 (61.9%)	22 (42.3%)	8 (32.0%)
Post-menopause		79 (49.1%)	32 (38.1%)	30 (57.7%)	17 (68.0%)
**Diabetes**	161	5 (3.1%)	1 (1.2%)	1 (1.9%)	3 (12.0%)
**Histology**	161				
Ductal		155 (96.3%)	81 (96.4%)	49 (94.2%)	25 (100.0%)
Lobular		4 (2.5%)	2 (2.4%)	2 (3.8%)	0 (0.0%)
Others		2 (1.2%)	1 (1.2%)	1 (1.9%)	0 (0.0%)
**Tumor size**	137				
T1		84 (61.3%)	45 (61.6%)	25 (56.8%)	14 (70.0%)
T2		46 (33.6%)	23 (31.5%)	18 (40.9%)	5 (25.0%)
T3		5 (3.6%)	3 (4.1%)	1 (2.3%)	1 (5.0%)
T4		2 (1.5%)	2 (2.7%)	0 (0.0%)	0 (0.0%)
**Subtype**	161				
Luminal A		69 (42.9%)	34 (40.5%)	25 (48.1%)	10 (40.0%)
Luminal B		41 (25.5%)	26 (31.0%)	12 (23.1%)	3 (12.0%)
HER2		27 (16.8%)	14 (16.7%)	5 (9.6%)	8 (32.0%)
Triple negative		24 (14.9%)	10 (11.9%)	10 (19.2%)	4 (16.0%)
**Breast density**	147				
Type A		10 (6.8%)	2 (2.6%)	1 (2.2%)	7 (29.2%)
Type B		25 (17.0%)	11 (14.3%)	8 (17.4%)	6 (25.0%)
Type C		81 (55.1%)	42 (54.5%)	28 (60.9%)	11 (45.8%)
Type D		31 (21.1%)	22 (28.6%)	9 (19.6%)	0 (0.0%)
**Cholesterol**	144				
Normal		69 (47.9%)	36 (48.0%)	21 (44.7%)	12 (54.5%)
High (≥ 200 mg/dl)		75 (52.1%)	39 (52.0%)	26 (55.3%)	10 (45.5%)
**Vitamin D**	133				
Low (< 20 ng/ml)		53 (39.8%)	29 (40.3%)	17 (39.5%)	7 (38.9%)
Normal (≥ 20 ng/ml)		80 (60.2%)	43 (59.7%)	26 (60.5%)	11 (61.1%)
**Insulin**	125	5.4 [3.9–7.2]	4.5 [3.3–6.0]	6.1 [4.4–8.5]	9.5 [5.7–10.8]

^a^Median [IQR]; n (%)

Hypertension and diabetes were present in 19.1% and 3.1% of patients, respectively; 49.7% reported doing exercise (more than 150 min per week), and 21% were smokers. All patients used olive oil in their diet and ate fruits and vegetables regularly (more than 5 days per week).

The median body weight was 65 [58–72] kg, the median height was 162 [157–167] cm, and the median abdominal circumference was 89 [81–95] cm.

At diagnosis, 84 patients [52%] had normal weight (BMI 18.5–24.9 kg/m^2^), 52 patients [32%] were overweight (BMI 25–29.9 kg/m^2^), and 25 [16%] were obese (BMI ≥ 30 kg/m^2^).

### BMI with molecular subtype and MBD analysis

**Fig. 1:**
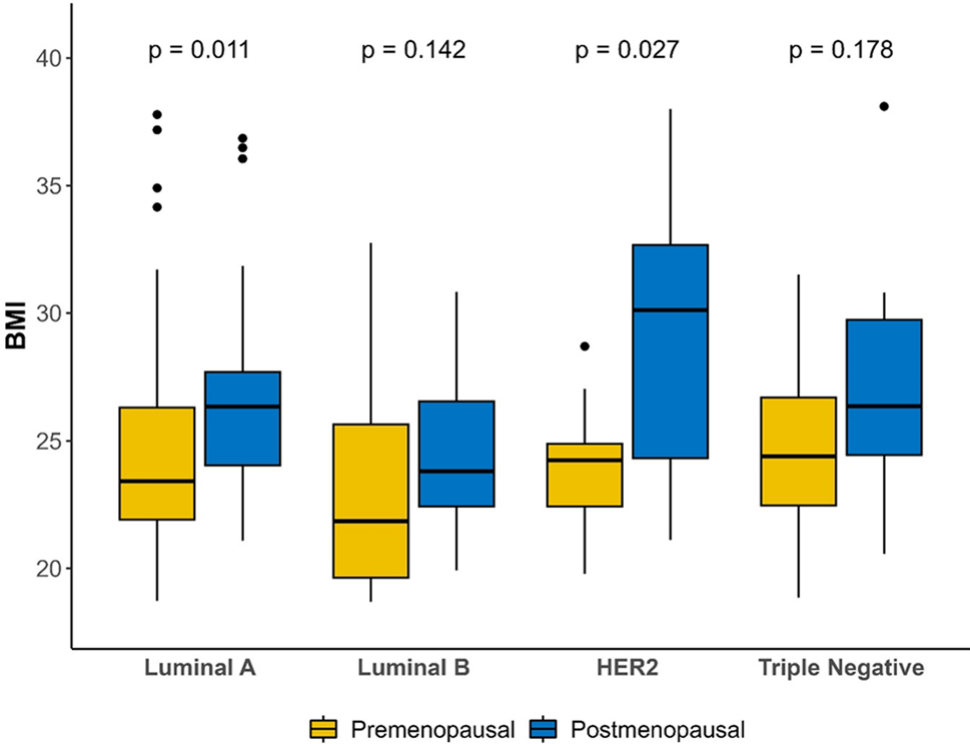
Molecular breast cancer subtype and BMI analysis according to menopausal status

No association was observed between BMI and molecular subtype, with higher BMI in postmenopausal women in all breast cancer subtypes. Analysis according to menopausal status found that BMI was significantly higher in postmenopausal than premenopausal patients in luminal A (*p* = 0.011) and HER2-positive (*p* = 0.027) subtypes (Fig. 1).

**Fig. 2:**
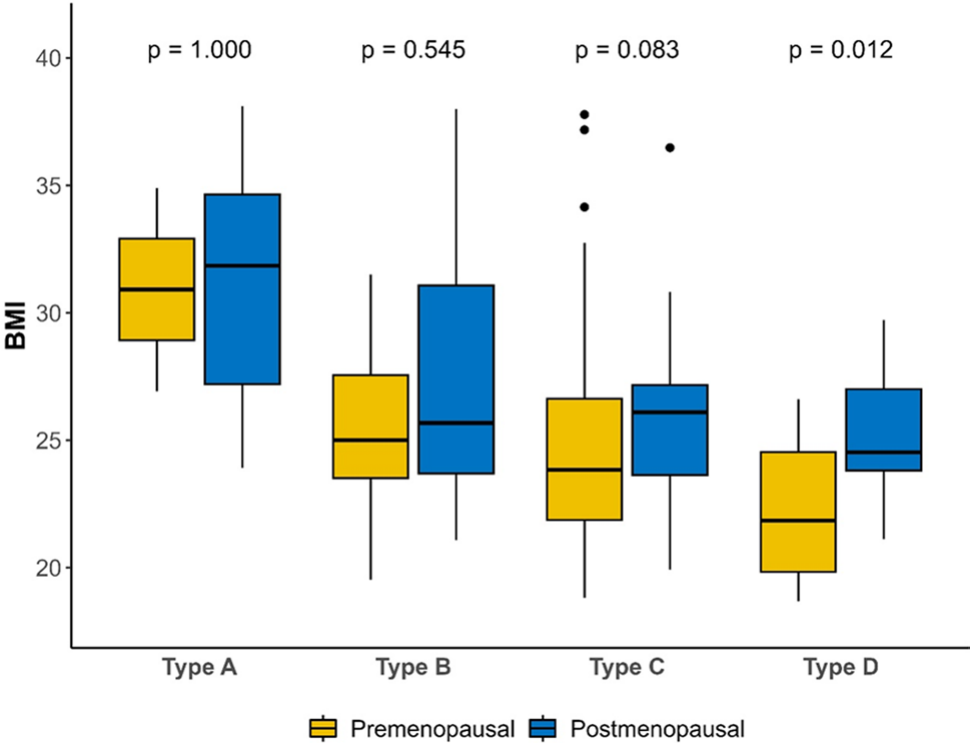
MBD and BMI analysis according to menopausal status

There was no association between MBD and molecular subtype, but significant differences between BMI and MBD (*p* < 0.001) were observed, with higher BMI in patients with type A density (“fat breast”) and lower BMI in patients with type D (“dense breast”). These differences were more relevant in premenopausal (*p* = 0.010) than postmenopausal women (*p* = 0.050). Furthermore, in patients with MBD type D, we found significantly higher BMI in postmenopausal than premenopausal patients (*p* = 0.012) (Fig. 2).

**Fig. 3:**
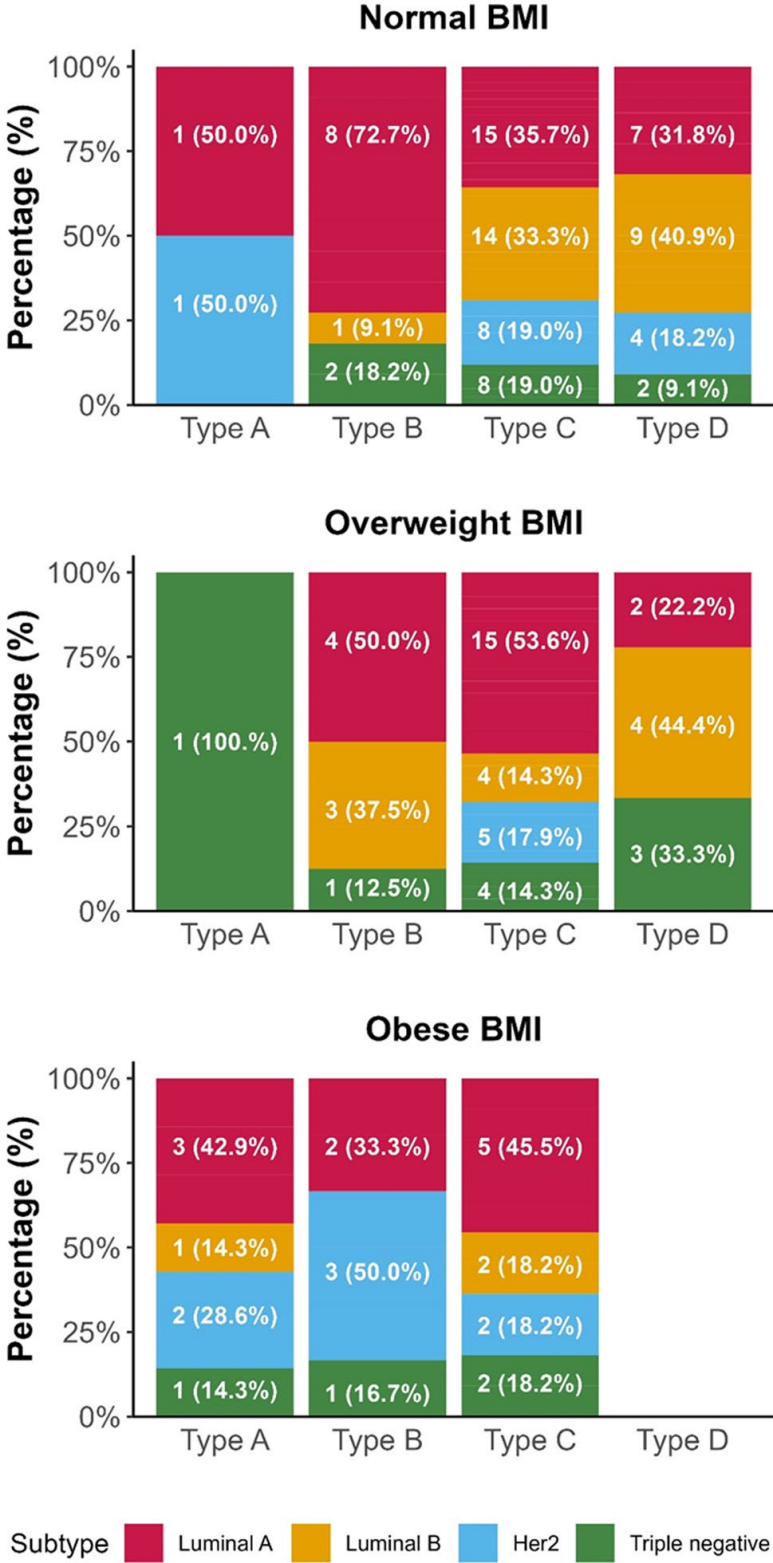
MBD and molecular subtypes according to BMI

Analysis of molecular subtype and MBD according to the three BMI groups (normal weight, overweight, and obese) showed no significant differences in the overall study population or according to menopausal status (Fig. 3).

### Cholesterol, vitamin D, and insulin analysis

**Fig. 4:**
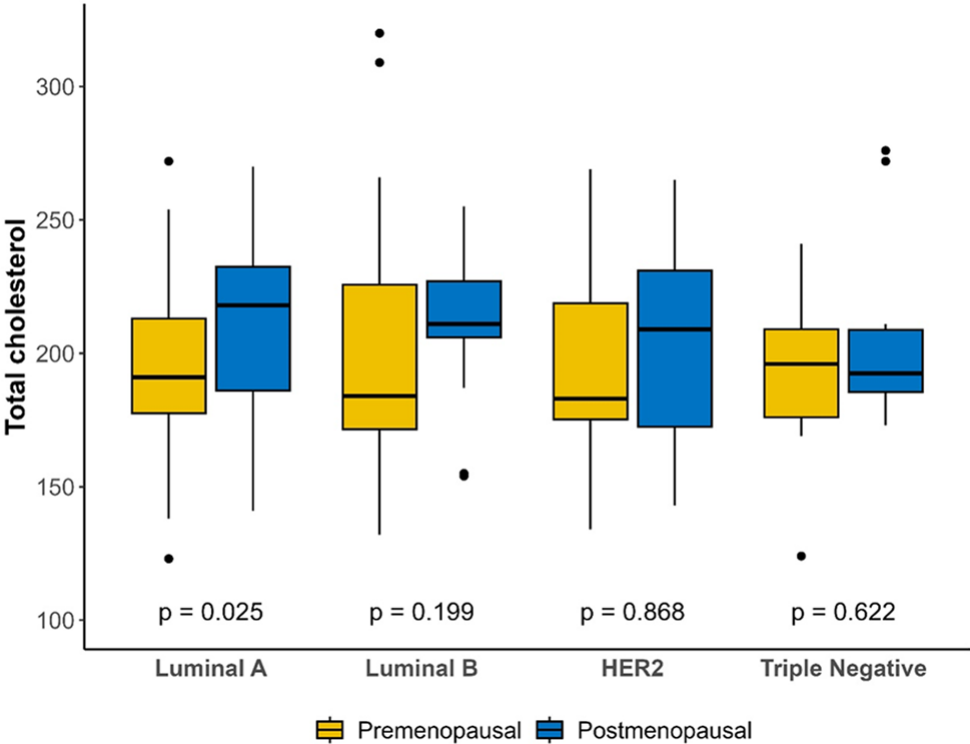
Molecular breast cancer subtype and cholesterol level analysis according to menopausal status

The overall analysis found no association between total cholesterol and MBD (*p* = 0.42), molecular subtype (*p* = 0.76), or BMI (*p* = 0.78), although there was an association between HDL-cholesterol and BMI: patients with higher BMI had lower HDL-cholesterol (*p* < 0.001) and this association remained independently of menopausal status. However, in the luminal A subtype, the total cholesterol level was significantly higher in postmenopausal than premenopausal patients (*p* = 0.025), with no significant differences in the other subtypes (Fig. 4).

There was no significant association between vitamin D and MBD, molecular subtype, or BMI.

A positive relationship was observed between insulin levels and BMI (*p* < 0.001), with higher levels of insulin associated with higher BMI, but not with MBD or molecular subtype, independently of menopausal status (Table 2).

**Table 2:** Cholesterol, vitamin D, and insulin analysis according to BMI and menopausal status

		Premenopausal		Postmenopausal	
		Spearman ρ	p-value	Spearman ρ	p-value
BMI	Total cholesterol	0.1	0.394	−0.21	0.08
	LDL-cholesterol	0.19	0.114	−0.052	0.67
	HDL-cholesterol	−0.31	** < 0.001**	−0.38	**0.001**
	Vitamin D	−0.1	0.416	−0.12	0.314
	Insulin	0.46	** < 0.001**	0.34	** < 0.001**

## Discussion

In the current study of women with early breast cancer, data showed that almost 50% of patients were overweight (32%) or obese (16%), which aligns with data for the general female population in Spain (30.6% overweight, 15.5% obesity) [25], but is lower than in other countries (USA, 37.3% obesity) [1]. Our findings are in line with previous reports on the association between increased BMI and higher breast cancer incidence in postmenopausal, but not in premenopausal, women [4–7]: 68% of obese patients were postmenopausal and 61.9% of patients with normal weight were premenopausal.

Only 7 patients (5.1%) in our study population were T3–T4, so we were unable to evaluate any association between tumor size and BMI. Engin et al. described more aggressive breast cancers with large tumor size, high-histological grade, and estrogen receptor-negative in patients with low adiponectin levels [26].

In the current study, 49.7% of patients reported doing exercise and 21% reported smoking; these data are similar to general population data for women in Spain with 54.8% of individuals reporting being sedentary and 20% being smokers [25].

We did not find any association between BMI and molecular subtype but, according to menopausal status, we observed higher BMI in postmenopausal luminal A and HER2-positive patients, as published in other studies that showed obesity as a risk factor for hormone receptor-positive breast cancer in postmenopausal women [2]. However, in contrast to previous reports [8, 9], we did not find a higher incidence of triple-negative breast cancer in obese/overweight premenopausal patients.

High MBD is an independent risk factor for breast cancer [11], and 76.2% of our patients had MBD type C or D (55.1% and 21.1%, respectively). Our study findings align with previous reports that MBD and BMI are independent risks factors of breast cancer [27] because, in our population, we observed decreased MBD with increasing BMI; this difference was more notable in premenopausal women, with high BMI in premenopausal patients with low MBD, and low BMI in premenopausal patients with high MBD. Furthermore, unlike other authors that described a higher risk of ER-negative breast cancer in premenopausal women with high MBD and high BMI [28], we found no association between MBD and molecular subtypes.

Half of our patients had hypercholesterolemia at diagnosis (52.1%), similar to data for the general Spanish population (50.5%) [29] and, unlike other studies [18, 19], we did not find any association between total cholesterol and MBD or more aggressive subtypes (triple-negative or HER2-positive); on the contrary, we found higher levels of cholesterol in postmenopausal patients with luminal A subtype. These findings suggest that total cholesterol may not be the most appropriate biomarker and future studies may need to assess 27-OH-cholesterol.

We were unable to study the potentially protective effect of vitamin D because we only collected data about vitamin D levels at diagnosis; 39.8% of patients had low vitamin D and there was no association with BMI. Ismail et al. described vitamin D deficiency in 30% of Egyptian females with breast cancer and an association with the HER2-positive subtype and worse prognosis [30].

In line with previous literature [24], we found that hyperinsulinemia was associated with obesity, with no differences according to menopausal status.

## Conclusions

This study, conducted in a population of women with a recent diagnosis of early breast cancer in Spain, showed higher BMI in luminal A and HER2-positive postmenopausal patients, and higher BMI in patients with low MBD regardless of menopausal status.

## Data Availability

The data that support the findings of this study are available from the corresponding author upon reasonable request.
